# Computational Protein Engineering: Bridging the Gap between Rational Design and Laboratory Evolution

**DOI:** 10.3390/ijms131012428

**Published:** 2012-09-28

**Authors:** Alexandre Barrozo, Rok Borstnar, Gaël Marloie, Shina Caroline Lynn Kamerlin

**Affiliations:** 1 Department of Cell and Molecular Biology, Uppsala Biomedical Center (BMC), Uppsala University, Box 596, S-751 24 Uppsala, Sweden; E-Mails: alexandre.barrozo@icm.uu.se (A.B.); rok.borstnar@ki.si (R.B.); gael.marloie@icm.uu.se (G.M.); 2 Laboratory for Biocomputing and Bioinformatics, National Institute of Chemistry, Hajdrihova 19, SI-1000 Ljubljana, Slovenia

**Keywords:** *de novo* enzyme design, enzyme redesign, protein engineering, directed evolution, computational enzymology

## Abstract

Enzymes are tremendously proficient catalysts, which can be used as extracellular catalysts for a whole host of processes, from chemical synthesis to the generation of novel biofuels. For them to be more amenable to the needs of biotechnology, however, it is often necessary to be able to manipulate their physico-chemical properties in an efficient and streamlined manner, and, ideally, to be able to train them to catalyze completely new reactions. Recent years have seen an explosion of interest in different approaches to achieve this, both in the laboratory, and *in silico*. There remains, however, a gap between current approaches to computational enzyme design, which have primarily focused on the early stages of the design process, and laboratory evolution, which is an extremely powerful tool for enzyme redesign, but will always be limited by the vastness of sequence space combined with the low frequency for desirable mutations. This review discusses different approaches towards computational enzyme design and demonstrates how combining newly developed screening approaches that can rapidly predict potential mutation “hotspots” with approaches that can quantitatively and reliably dissect the catalytic step can bridge the gap that currently exists between computational enzyme design and laboratory evolution studies.

## 1. Introduction

Since Friedrich Wöhler first synthesized urea in 1828 [1], organic chemists have worked on synthesizing a breathtaking range of organic compounds that play a role in all aspects of modern life, from food preservation to dyes, pharmaceuticals and fuels. Due to their ubiquity, a great variety of artificial catalysts have been created to accelerate organic synthesis, from compounds using simple acid/base catalysis to organometallic catalysts using transition metals such as palladium [2], platinum [3], iridium [4], rhodium [5] or gold [6] as their metal centers. Particularly, the use of transition metals has permitted us to increase our ability to form carbon-carbon bonds [7–9], a process which forms the basic building block of almost all synthetic organic chemistry. However, such progress has not been without its challenges. That is, in addition to cost concerns, many industrial processes harnessing such catalysts occur under environmentally unfriendly conditions that go against the important current emphasis on “green chemistry”, which encourages sustainable development [10].

In principle, enzymes should provide the perfect solution to these problems. As one example, the half-life for the hydrolysis of an amide bond is in the range of hundreds of years (the half-life for the hydrolysis of a glycine-glycine dipeptide in neutral solution at 25 °C is 350 years [11]). If the same dipeptide is transferred to 1 M HCl or NaOH solutions (at the same temperature), the half-life for the reaction drops to approximately 150 and 2 days, respectively [12]. While this is of course impressive, under enzymatic catalysis, the reaction rate is increased by a factor of 10^12^ [11,12] (and there are many examples of enzymes facilitating even more extreme rate enhancements, see e.g., [12,13]). Such proficiencies have never been matched in a man-made catalyst (although changing the reaction medium from water to alcohol has been demonstrated to produce enzyme-like rate enhancements in the case of dinuclear zinc catalysts of phosphate diester hydrolysis [14]). In addition to their extreme proficiencies, they are also biodegradable and reusable catalysts [15], making them ideal green reagents. Also, while most enzymes work within limited and tightly controlled temperature ranges, there do exist enzymes that can act anywhere within a temperature range from 0 to 100 °C (for the two extremes observed in extremophile bacteria) [16]. On top of this, enzymes have chiral active sites, making them able to discriminate between different stereoisomers and regioisomers, with at times quite high efficiency, making them ideal catalysts for enantio- and regioselective chiral chemistry, in order to generate isomerically pure pharmaceuticals and fine chemicals [17,18].

The challenge is that the same properties that make enzymes such proficient catalysts are directly orthogonal to the needs of biotechnology [19]. That is, enzymes have had millions of years in order to evolve to become the proficient catalysts that we see today, and they need to operate under tight *in vivo* regulation. As a result of this, they are picky about what substrates they are willing to accept (which can be limited by both electrostatic, and simple steric considerations), their efficiencies vary dramatically for different substrates [20], and, since they need to be turned off as well as on, often they can be inhibited by even their own product [21]. They are also often highly resistant to environments other than the one they have evolved for. In contrast, biotechnology needs catalysts that can work under harsh conditions if necessary, tolerate changes in environment, catalyze a broad range of substrates to generate maximal amounts of product, and, ideally, can catalyze completely new reactions for which no catalyst currently exists. Therefore, in order to fulfill these requirements, it is often not sufficient to take enzymes “as is”, but rather, it is necessary to be able to modify their physico-chemical and functional properties in a fast, efficient and streamlined manner. In light of this, it is perhaps unsurprising that interest has exploded in engineering enzymes for biocatalysis, and, as of 2012, that this area is one of the fastest growing of the biotechnology sector [22].

While it could be argued that “biocatalysis” dates back thousands of years to Sumerians and Egyptians using microorganisms to produce beer, the development of techniques such as error-prone PCR [23] and DNA shuffling [24] have essentially revolutionized biocatalysis [25], as they allow enzyme properties to be altered through random mutagenesis, in a “directed” fashion (directed evolution, DE), where the enzyme is iteratively refined until a property of interest (such as, for instance, a specific function or improved selectivity towards a given enantiomer) is observed. However, despite the tremendous power of this approach and the advances it has allowed in protein engineering, it is still subject to a number of limitations, the most pressing of which is the sheer vastness of the sequence space that needs sampling. That is, a simple enzyme comprised of 300 amino acids will have a sequence space of 20^300^ possible mutations (if one uses the naturally occurring building blocks alone), which is far out of the reach of even the best synthetic libraries [26,27]. This problem is further compounded by the fact that, in a random library, the frequency of a beneficial mutation occurring is only ~10^−3^ [28,29], whereas that of a deleterious mutation is ≥0.33 [30]. Various approaches have been developed to deal with this issue, including approaches such as iterative saturation mutagenesis [31] and simultaneous multiple-site saturation mutagenesis [32], which focus on limiting the library sizes necessary to effectively obtain enhancements in activity, or even using ancestral libraries [26] (to name a few examples). However, clearly, as the rounds of directed evolution progress, most mutations quickly become unviable, and the library sizes that need managing become simply astronomical. In addition to this, while one of the strengths of using directed evolution is that, in principle, little-to-no knowledge of the actual catalytic mechanism being employed by the enzyme is required in order to be able to obtain substantial catalytic enhancements, this also poses a weakness, as without insight into why DE mutants are catalytic (or anticatalytic) it becomes challenging to further improve and refine the selection procedure for a given property.

As there are a large number of reviews addressing the advantages and challenges of laboratory-based evolution techniques for enzyme optimization [33,34], we will not be addressing this issue in the present work. However, computers have the potential to play a major role in guiding and directing experimental work, and increasing realization of this fact has led to an explosion in activity in this area. While computational enzyme design could still be classed as a nascent field, recent years have seen major advances in a broad spectrum of areas, from (computational) enzyme redesign, to the *de novo* design of enzymes from the basic building blocks upwards. This review will outline some recent developments in this field, introducing different “philosophies” towards computational enzyme design, and present suggestions for important future directions. Our focus will be on improving functional properties of enzymes rather than their physico-chemical properties such as thermostability and solubility, which are out of the scope of the present review. Specifically, we will illustrate the fact that, when combined with ever-increasing computational power, current computational approaches are rapidly reaching a stage where it is possible to rationally mimic the process of laboratory evolution *in silico*, bridging the gap that exists between the proficiencies of artificially designed catalysts and the outcome of laboratory evolution studies.

## 2. “*De Novo*” Enzyme Design

**Figure 1:**
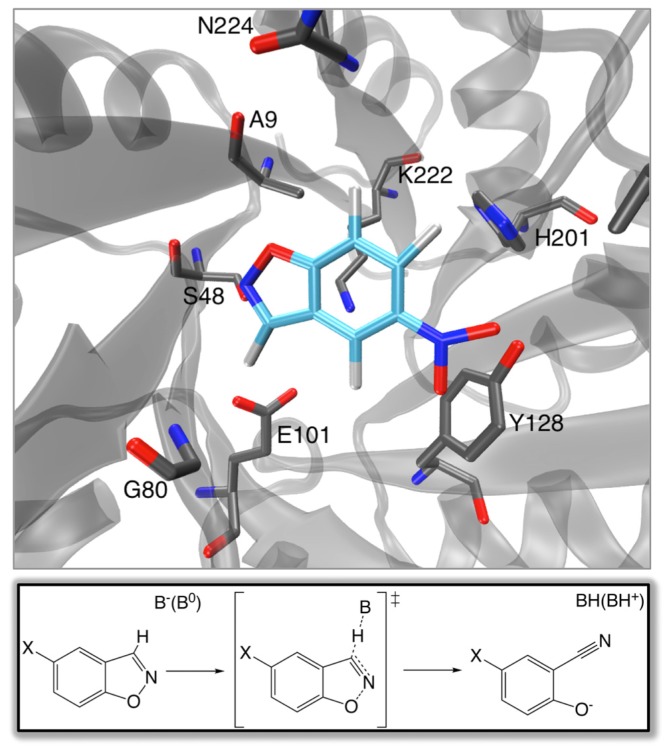
Model of the active site of a representative *de novo* catalyst of the Kemp elimination reaction [43] (shown in its generalized form in the inset below). Note the position of E101, which was introduced to act as a general base, relative to that of the substrate. However, it is important to point out that the design was executed relative to the transition state and not the native substrate, and, therefore, the contacts in this figure are not truly idealized.

One of the “Holy Grails” of biotechnology would be the ability to rapidly design effective biological catalysts for new chemistry completely from scratch on demand. This goal was one step closer to realization in 2008, with the computational design of eight enzymes capable of performing base-catalyzed benzisoxazole ring opening (Kemp elimination) with rate accelerations of ~10^5^, and multiple (≥7) turnovers [35], although this is similar to the rate in solution when the cost of bringing the reacting fragments into the “reacting cage” is taken into account (see [36,37]). Catalysis of this reaction, which does not occur in nature, is well characterized, and has been performed by systems ranging from catalytic antibodies [38,39], to “off-the-shelf” proteins such as bovine (and other) serum albumens [40,41], to small synthetic enzyme mimics made of organic molecules (“synzymes”) [42]. The successful design of *de novo* enzymatic catalysts for this reaction was achieved using a multi-level approach [35]. A first port of call was the design of an idealized active site for benzisoxazole ring opening, which incorporated the necessary catalytic machinery for general base catalysis, as well as a number of other residues necessary for efficient chemistry [43] (see Figure 1). This was achieved by constructing an appropriate “theozyme”, which optimizes relevant functional groups necessary for efficient chemistry around a proposed transition structure based on quantum mechanical calculations [44]. The next step was to match this theozyme to a suitable scaffold, which was done using the Rosetta Match hashing algorithm [45]. A search through a scaffold set containing >100,000 members, which included multiple protein folds such as β-propellers, jelly rolls, Rossman folds and lipocalins ultimately favored a TIM barrel (which is also a widespread fold in naturally occurring enzymes). Subsequent experimental characterization of this design demonstrated an r.m.s.d. deviation of <1 Å (full backbone plus active site sidechains) between the designed and crystallographic structures, with rate accelerations (*k*_cat_/*k*_uncat_) up to 10^5^ as outlined above, and *k*_cat_/*K*_M_ values up to 163 M^−1^ s^−1^, making it impressive that it was possible to design an effective catalyst using this approach. Nevertheless, the resulting catalytic efficiencies still range far behind that of catalytic antibodies or serum albumens [41], although still presenting progress on elegant earlier work that used computational search algorithms to identify catalytically active enzyme-like active sites in protein scaffolds [46]. Initial *in vitro* evolution was able to modestly increase the catalytic activity of the designed enzymes to provide rate accelerations in the region of factors of 10^4^ to 10^6^ (*i.e.*, a *k*_cat_ value of 1.37 s^−1^). This is a 75-fold increase over the initial designed variant used as a starting point in this work, which had a *k*_cat_ of 0.018 s^−1^ [35,47]). In addition, the *k*_cat_/*K*_m_ value was improved to 2590 M^−1^ s^−1^ (compared to 12.2 M^−1^ s^−1^ in the initial design [35,47]), after the introduction of eight mutations over seven rounds of evolution, showing the evolvability of the designed systems. Specifically, these initial mutations were determined to act by tuning the electrostatic environment of the active site, as well as correcting a problem in the initial design, which introduced a lysine as a general acid for leaving group protonation, but placed it such that it’s pK_a_ would be detrimentally shifted by “quenching” from the catalytic base. Subsequent combined experimental and theoretical work [48] on a different (and more active) designed enzyme, KE70 [35] (*k*_cat_ of 0.14 s^−1^ and *k*_cat_/*K*_M_ of 126 M^−1^ s^−1^), managed to further fine-tune the active site electrostatics, redesign the active site in order to improve substrate binding, as well as stabilize a catalytic His-Asp dyad in an optimal catalytic conformation. This, in combination, resulted in a >400-fold improvement in catalytic proficiency (for practicality, we distinguish here between catalytic enhancement (or efficiency) and proficiency, where by catalytic enhancement (or efficiency) we refer to the rate of the catalyzed *versus* the uncatalyzed reactions (*i.e.*, *k*_cat_/*k*_uncat_), whereas by proficiency we refer to the overall *k*_cat_/*K*_M_ value.), with *k*_cat_/*K*_M_ values of as high as 5 × 10^4^ M^−1^ s^−1^ in the best variants [48], and a ~75-fold increase in *k*_cat_ compared to the original design. A more recent work [49] has also introduced consensus mutations into one of the best of the original designs [35] (*k*_cat_/*K*_M_ ~ 160, *k*_cat_ was not measurable due to instability of the design), and screened for enzymatic activity with a number of substrates (5,7-dichloro, 6-chloro and 6-fluoro benzisoxazole), none of which showed activity above the detection limit. In all cases, however, after 16 rounds of mutation, not only were activities observed, but also, in the case of 5,7-dichloro substituted benzisoxazole, a *k*_cat_ of 21.2 s^−1^ and a *k*_cat_/*K*_M_ of 573,090 M^−1^ s^−1^ was observed. Now while Kemp elimination presents something of a “test system” for artificial enzyme design, nevertheless, it provides a good proof-of-concept of the fact that it is possible to start from a minimal, idealized active site, and match this to a pre-existing scaffold to end up with an active (albeit inefficient) catalyst, that can be further improved by subsequent laboratory evolution.

**Figure 2:**
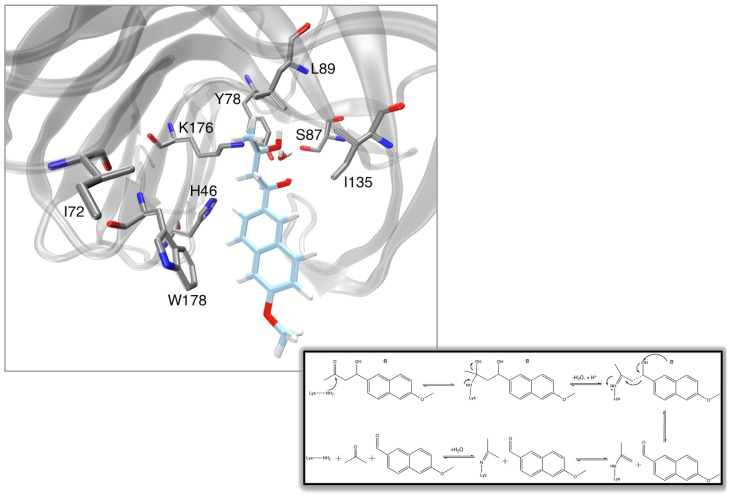
Model of the active site of a representative *de novo* designed retroaldolase [50], with a generalized version of the aldol reaction pathway using a lysine nucleophile and acid-base catalysis shown in the inset. Note that the figure depicts the enzyme in complex with the native substrate, and *not* with the transition state, and therefore the contacts are not truly idealized.

A number of other examples of *de novo* enzyme design have been observed in the literature recently. For instance, the approach used in [35] was expanded in order to design an active site that can accommodate multiple TS and reaction intermediates during the course of multistep reaction pathways. Specifically, a catalyst was designed *de novo* that was capable of facilitating the cleavage of carbon-carbon bonds [50] (a reaction which is fundamental to organic chemistry) with multiple turnovers and modest rate accelerations of up to four orders of magnitude (modest again in comparison to accelerations routinely obtained by natural systems) in the designed variants. An overview of the retro-aldol reaction catalyzed by this enzyme, as well as a schematic of a representative designed active site is shown in Figure 2. As with previous work [35], an initial theozyme was constructed, which harnessed two lysines close to each other in order to reduce the pK_a_ of the nucleophilic lysine, as well as an aspartate which allows for general base catalysis. This was then coupled to an exhaustive search of 181,555 distinct protein scaffolds that could accommodate this theozyme, which resulted ultimately in 72 designs that were selected for experimental characterization. Of these, 32 showed detectable activity [50] (*k*_cat_ values up to 9.3 × 10^−3^ s^−1^, *k*_cat_/*K*_M_ values up to 0.74 M^−1^ s^−1^). Once again, this is not a highly proficient catalyst, however, as a proof-of-concept study of the possibility for designing catalysts from scratch for complex chemical reactions, this is elegant work. Subsequent analysis of the designed systems [51] suggested that the observed catalytic enhancements come from a number of sources, most importantly the effect of the enzyme environment on lowering the pK_a_ of the catalytic lysine, as well as hydrophobic binding interactions in the enzyme active site. It was therefore suggested that improvements in binding interactions and the placement of catalytic groups could improve design efforts, a suggestion compounded by more recent studies into the interaction of these enzymes with covalent enzyme-substrate analog complexes [52]. Once again, site directed mutagenesis and laboratory evolution could be applied to the designed retroaldolases [53], extending on the catalytic motifs used in the original work [50], allowing for increases of up to 0.71 min^−1^ for *k*_cat_, and 490 M^−1^ s^−1^ for *k*_cat_/*K*_M_.

**Figure 3:**
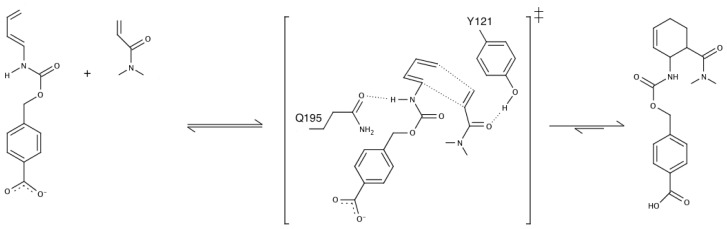
The Diels-alder reaction, showing a pericyclic (4 + 2) cycloaddition to form a chiral cyclohexene ring. Also illustrated here are the residues selected to act as donor and acceptor in the designed Diels alderase discussed in the main text [54].

A third recent example of *de novo* enzyme design is the computational design of a biological catalyst for a stereoselective bimolecular Diels Alder reaction [54]. The overall reaction mechanism and a representation of the designed active site are shown in Figure 3. This system is significant, as there is no natural counterpart capable of catalyzing the Diels-Alder reaction. The fact that it can catalyze the formation of two carbon-carbon bonds to stereoselectively yield four new stereogenic centers is of great importance to organic chemistry, although now the catalytic proficiency is even poorer for this challenging reaction, with *k*_cat_ values of at most 2.13 h^−1^, presenting a modest rate acceleration of only 12-fold compared to the uncatalyzed reaction at 298 K [54]. However, to achieve this modest acceleration, the designed variant uses a clever chemical trick. Specifically, the Diels-Alder reaction is under orbital control, with the reaction rate depending on the gap between the highest occupied molecular orbital (HOMO) of the diene, and the lowest unoccupied molecular orbital (LUMO) of the dienophile. Therefore, in the designed variant, two hydrogen bonds were introduced to increase the HOMO of the diene and decrease the LUMO of the dienophile, respectively, and these between them, if correctly positioned, were expected to provide ~4.7 kcal/mol transition state stabilization (based on quantum chemical calculations of the theozyme [54]), although, as mentioned above, the actual observed catalytic enhancement was much smaller.

**Figure 4:**
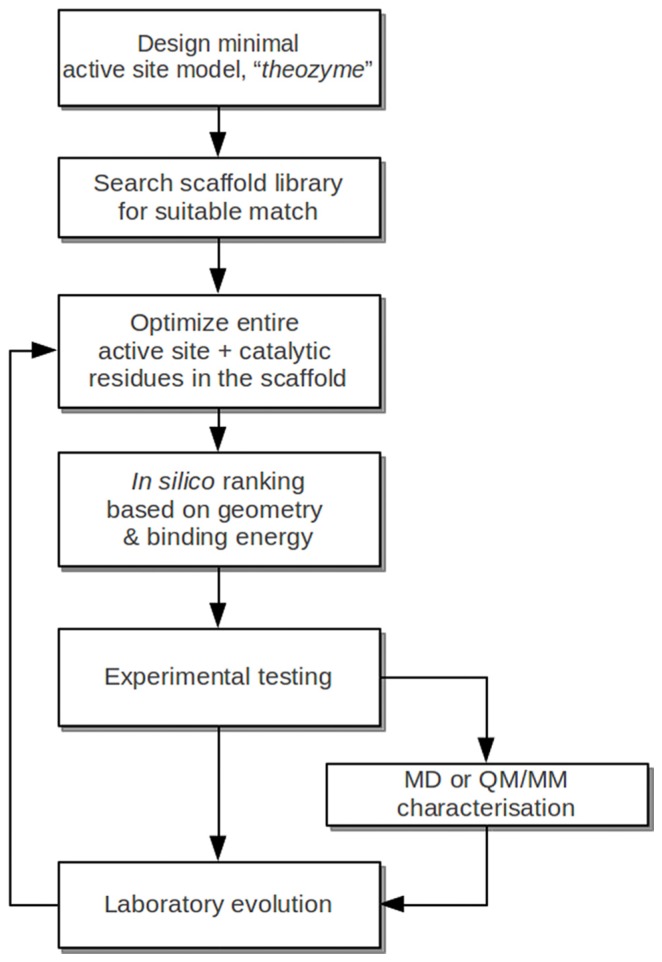
A generalized version of a currently popular protocol for *de novo* enzyme design, adapted and abridged from [50]. Note that while molecular dynamics (MD) or quantum mechanical/molecular mechanical (QM/MM) characterization was not explicitly included in [50], it has been successfully used to aid the design process.

**Figure 5:**
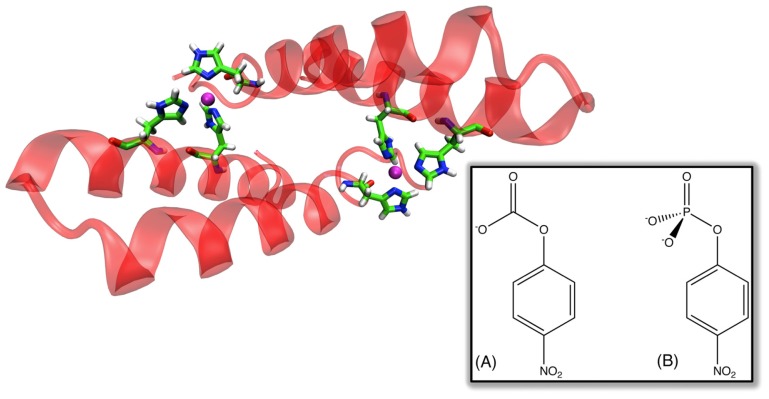
Structural overview of a computationally designed zinc-mediated protein interface [57], that (unintentionally) is capable of promiscuously catalyzing both (**A**) *p*-nitrophenyl acetate (*k*_cat_/*K*_M_ = 630 M^−1^ s^−1^) and (**B**) *p*-nitrophenyl phosphate hydrolysis (*k*_cat_/*K*_M_ = 14 M^−1^ s^−1^).

We have presented here a few examples of successful *de novo* enzyme design, which have focused on using the generalized protocol illustrated in Figure 4. This is a field that has grown rapidly in interest, and space considerations prevent us from discussing all recent work in similar detail, and there have been other recent reviews that address this issue (e.g., [54], as one example). However, some works that we would like to mention here include a recent work by Faiella and coworkers, in which not only the active site, but also the scaffold was designed from first principles [55]. This resulted in an artificial di-iron oxo-protein, with phenol oxidase activity and, depending on substrate, *k*_cat_ values of up to 13.5 min^−1^, and *k*_cat_/*K*_M_ values of up to 6315 M^−1^ min^−1^ (for the best substrates). Another recent example comes in the form of a computationally designed homodimeric zinc-mediated protein interface [56], which was originally designed in order to study protein-protein interactions, but has recently been demonstrated to be a promiscuous catalyst capable of at least two functionalities [57] (Figure 5), namely the hydrolysis of *p*-nitrophenyl acetate and phosphate, with rate accelerations of 10^5^ and 10^4^-fold, as well as *k*_cat_/*K*_M_ values of 630 and 14 M^−1^ s^−1^, respectively. This is noteworthy in light of the fact that this system was not designed with catalytic ability in mind, but in comparison to many designed enzymes it shows surprising potential as a catalyst, opening other interesting avenues in protein engineering using simple model systems. Clearly, the works presented here demonstrate that we are moving closer to being able to design effective enzymes from scratch. However, there are still a number of significant issues. As will be discussed in the Sections 4 and 5, any rational design strategy requires in-depth knowledge of an enzyme’s catalytic mechanism, making it very difficult to predict how an enzyme will behave *a priori*, as was illustrated in the example of the pK_a_ quenching problem in the designed Kemp eliminase (see [35,47]). Additionally, current design strategies such as those highlighted above cannot in and of themselves distinguish between active and inactive constructs, and therefore subsequent testing is left to experiment. The third issue is that, at present, the efficiencies of *de novo* designed systems are far too low to be useful in commercial settings and can only be improved to come closer to that of natural enzymes following subsequent laboratory evolution. While this has led to significant improvements in activity, this will ultimately always be limited to the same challenges all directed evolution studies face, namely an inability to explore the entire sequence space and library sizes. Therefore, for such designs to ultimately be effective, approaches will be needed to bridge the gap between *in silico* design and laboratory evolution. Therefore, Section 3 will address advances in and insights obtained from rational enzyme design, and Section 4 will address machine-learning approaches and novel screening approaches that can be used to greatly aid the design process.

## 3. Computational Enzyme Redesign

As was seen in Section 2, recent years have seen tremendous advances in our ability to design enzymatic catalysts for novel chemistry “from scratch”. However, what about the enzymes that Nature has already provided? A wide variety of chemical reactions are necessary to facilitate life, and, as a result, catalysts have evolved for a range of reactions from group transfers, isomerization and hydrolysis reactions to oxidation/reduction reactions. Therefore, many templates already exist that can, theoretically, be modified for desired chemistry (as an example, compare the artificially designed retroaldolase [50] presented in Section 2 with natural aldolases such as DERA from *E-coli*, which has been evolved successfully for industrial biocatalytic applications e.g., [58]). In view of this, it is perhaps unsurprising that there has been interest in not just experimental [59,60] but also computational [61,62] protein redesign for several decades. Despite many promising studies, (rational) computational protein redesign of functional properties is not without its challenges, as it requires a reliable 3-D structure of the system of interest, as well as in-depth insight into the catalytic mechanisms, which can be changed by mutations. Additionally, in cases where high-quality crystal structures of the enzyme *do* exist, the fleeting nature of transition states means that the best one can hope for are complexes with transition state analogues [63]. While these can carry useful structural information in terms of potential contacts, they can also be deceptive in terms of the information they provide as to the actual chemistry [64,65] and need to be treated with care. As a result of this, the enhancements obtained using rational design approaches have been modest, particularly in comparison to the proficiencies of naturally occurring enzymes [66]. However, as with *de novo* enzyme design, increasing computer power has greatly expanded the range of possibilities with respect to protein redesign, both in terms of approaches to qualitatively and quantitatively probe and modify activity, as well as in terms of computational approaches to rapidly identify potential mutations that can affect enzyme function and stability. In this section, we will illustrate and discuss some noteworthy recent applications of computational enzyme design, whereas Section 3 will focus on screening approaches that can bring us closer to *in silico* directed evolution becoming a reality.

Returning to the Kemp eliminases presented in Section 2, there have been a number of recent studies that have addressed the problem of computationally predicting and improving the catalytic activity of the designed enzymes (and closely related systems). Initial mixed quantum mechanical/molecular mechanical (QM/MM) and free-energy perturbation (FEP) calculations [67] that aimed to explore the energetics and mechanism of the reaction catalyzed by a number of the designed constructs as well as the background reactivity in water predicted activity in three of the examined constructs. In the fourth case, the authors obtained significant product trapping, however, uncertainty in the protonation states of a number of active site residues made results on this system inconclusive. In all enzyme-catalyzed cases, the authors obtained concerted mechanisms proceeding through a single transition state in which the proton transfer was quite advanced in comparison to the breaking of the isoxazolyl N–O bond (for the reaction mechanism, see Figure 1). A similar mechanism was obtained when modeling the background reaction in aqueous solution using a hydroxide base. This led the authors to suggest that, since the enzymes do not alter the mechanism compared to solution, design studies should focus on further increasing the basicity of the catalytic base as well as optimizing the positioning of catalytic residues in the active site [67]. It should be noted, however, that this work provided poor quantitative accuracy, with large discrepancies between calculated and observed catalytic effects. Subsequent work has used a range of computational approaches [68], including quantum mechanical cluster models comprising a smaller subset of catalytic residues, QM/MM calculations that take into account the full system, as well as MD simulations that explore structural changes to explore the origins of activity in designed systems. As the authors acknowledge, the fact that the cluster and QM/MM models do not account for protein reorganization as well as changes in solvent accessibility along the reaction coordinate made these approaches insufficient to distinguish between active and inactive designs. However, performing molecular dynamics simulations allowed for exploration of structural features in cases where the activity or inactivity was known, leading to the identification of “design flaws” causing inactive enzymes [68].

While such simulations are interesting and informative in the context of providing valuable structural insight, for effective enzyme redesign, it would be useful (and, indeed, most likely critical) to also have quantitative insight into the precise molecular basis for the observed catalytic (or anticatalytic) effect of different mutations, as well as, ideally, also, information about the quantitative contribution of different residues to activity and stability. To the best of our knowledge, most currently available quantum chemical approaches do not provide sufficient breakdowns of different contributions in order to be able to probe this issue, with the exception of a few notable examples such as the activation strain model [69], which provides a breakdown of the total energy intro contributions from electrostatic interactions, Pauli repulsion, and orbital interaction energies which could be used to dissect interactions between the reacting atoms (although even such elegant approaches become challenging in cases where one aims to explore the enzyme contributions, where using the linear response approximation with major sampling is to obtain reliable results, as demonstrated in [70]). Additionally, a second problem is computational cost: while, in principle, *ab initio* quantum mechanical approaches provide better precision, the cost involved in obtaining this precision makes it simply intractable to test numerous large systems, multiple mutations, different potential mechanisms and substrate binding modes, creating a major bottleneck in the design process.

Here, in our opinion, the empirical valence bond (EVB) approach of Warshel and coworkers [71–73] provides the perfect solution to these problems. The EVB approach is a semi-empirical QM/MM approach, based, as the name suggests, on the valence bond theory, which describes chemical reactivity by mixing resonance states corresponding to classical valence-bond structures for different possible reacting states and obtaining the free energy for moving between these using free-energy perturbation/umbrella sampling (FEP/US) [71,74]. It is important to point out that, despite the concerns that may arise from the fact that the EVB approach sacrifices precision due to the fact that it is semi-empirical, all quantum chemical approaches use approximations to varying extents, and, for instance, the inherent errors in commonly used DFT approaches can be quite significant (see discussion in e.g., [75] and references cited therein), and the reliability of the results are heavily dependent on the precise functional and basis set used. However, this becomes partially irrelevant, since the EVB accuracy is mainly in determining relative free energies (with respect to a well-defined reference state), or catalytic effects. Therefore, while it is in principle fully feasible to increase the precision of the EVB using approaches such as the paradynamics approach [76], unless one has the computational resources to perform quantum chemical calculations at a very high level of theory, any inherent error introduced through using empirical force fields is not greater than that existing in most commonly used DFT approaches (once the force field has been rigorously and carefully parameterized), particularly as an important core point of the EVB approach is careful calibration to not just *ab initio* but also experimental data available on the reference reaction in water. The fact that precision should not be a concern to the reader can be illustrated in the repeated success of the EVB approach at reproducing catalytic effects in wild-type and mutant enzymes with very high levels of quantitative accuracy, and without the need for adjustable parameters [13,36,37,77]. The strength of the EVB approach as a tool for computational enzyme redesign lies in three key features: (1) it’s relative speed, in that while it is still computationally demanding to perform the extensive sampling required to obtain physically meaningful convergent free energies, it is much faster than doing the same at a higher level of theory without compromising precision; (2) the tremendous amount of chemical information it carries as well as the energy-gap reaction coordinate, which allows for a reliable description of bond-breaking and bond-making events, while the energy-gap coordinate reduces the time required to obtain convergent results compared to other currently popular approaches, as recently shown in extensive benchmarks by Fuxreiter and coworkers [78]; and (3) the fact that the EVB approach is based on calibration to the correct reference reaction in solution, such that one is directly examining catalytic effects, and that it provides a detailed breakdown of such effects, allowing one to pinpoint the precise molecular basis for the observed catalytic effect. In combination, these approaches make the EVB ideal for not just understanding enzyme catalysis, but also as a tool for rapid predictive protein redesign, due to its ability to predict mutational effects with high accuracy.

**Figure 6:**
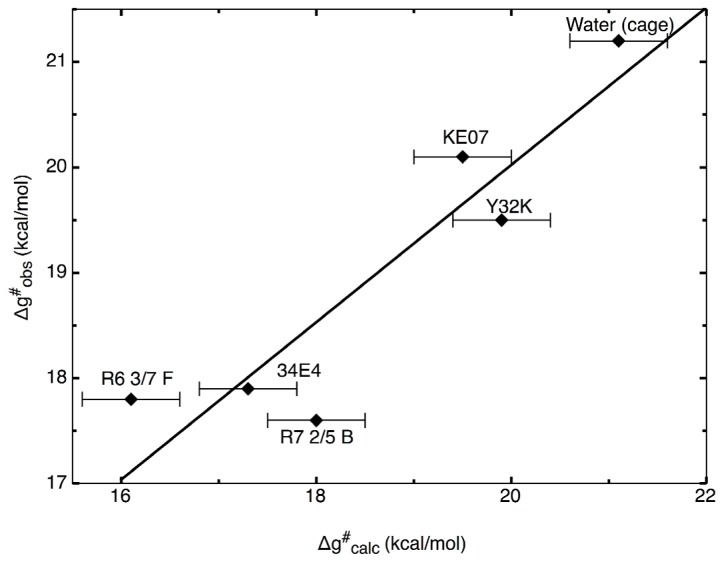
Correlation between calculated (Δg^≠^_calc_) and observed (Δg^≠^_exp_) activation barriers for the catalysis of the Kemp elimination reaction (illustrated in Figure 1) for a range of catalysts, including two catalytic antibodies (34E4 and Y32K), and a number of designed proteins in wild-type and mutant form (KE07, R6 3/7F and R7 2/5B). This plot is based on data presented in Table 1 of [37]. Calculations were performed using the empirical valence bond (EVB) approach, and, in all cases, both the difference between the calculated and experimentally observed values, as well as the standard deviation in the calculated values (over 20 trajectories) was <1 kcal/mol.

To illustrate the power of the EVB approach in protein redesign, we refer the reader to detailed EVB studies [36,37] of catalysis of the Kemp elimination shown in Figure 1. The first issue that we would like to highlight here again is the aforementioned one of precision. Namely, in these works, the authors first examined the molecular basis for catalysis of the Kemp elimination reaction in a wide range of systems, from catalytic antibodies through to serum albumens, and also computationally designed Kemp eliminases (KE) and designed variants. As can be seen from Figure 6, the authors were able to reproduce the catalytic effect of all known systems with very high accuracy (maximum difference between calculated and experimental activation barriers being within ~1 kcal/mol in approximately 20 tested systems [36,37] with standard deviations of maximum 1 kcal/mol over up to 20 trajectories).

Having established the reliability of the computational procedure, the authors then provided a number of significant insights into the reason for the observed catalytic effect of the studied mutants, as well as the challenges involved with obtaining proficient catalysis of this system. Specifically, it was observed that, in contrast to natural systems, which achieve efficient catalysis through stabilizing the transition state of the reaction, the mutant KEs are achieving enhanced catalytic activity through *destabilizing* the ground state of the reaction [36,37]. In principle one could argue that exactly how the catalysis is being achieved is not that significant as long as one can improve the efficiency of the enzyme. However, not only is this not observed in native enzymes [13], but also, the designed and evolved KEs are also not taking optimal advantage of this effect. This is important in light of suggestions that one should target the catalytic base [67], since, as discussed in [37], even if it were possible to create strong desolvation for the base in the ground state, this would result in a very large pK_a_ that would not help at physiological pH as the base would simply be protonated by the bulk. The second challenge that ties in with this is that the charge change between the ground state and transition state for this reaction is very small, making it extremely difficult to exploit active site polarity in order to better catalyze this reaction [36] (and, as was observed by [36,37], as a result, mutations that tend to effect activity also tend to be farther from the active site). This could also explain to some extent the current problems with trying to significantly improve this system, whether computationally or experimentally.

To address this issue, a number of screening approaches were presented [36,37] that could be used to screen for potential mutation hotspots (which could then be tested by EVB), as well as to predict stability change upon mutation, however, we will leave the discussion of these to Section 4, which deals with currently available approaches for performing *in silico* directed evolution. Here, we would just like to comment on a recent work [79] that used iterative MD and experimental analysis to explore previously inactive artificial KEs and used insights from MD and structural analysis to improve activity. We agree with the authors of this work [79] that an iterative procedure is important, as well as the fact that understanding inactive designs can provide insights that can potentially guide future design effort by avoiding repetition of “mistakes” that were made during the laboratory evolution (or initial computational design). However, this brings us back to the question at the end of Section 2: Considering the wide variety of templates Nature already provides, what is the best starting point? Here, we believe that the most effective approach for redesign, regardless of the starting point, is one which can provide quantitative as well as qualitative insight (such as that of [36,37]), which allows for the identification of factors that can be rapidly modified, and which can then be combined with an iterative approach such as that illustrated in [79] for rapid redesign.

**Figure 7:**
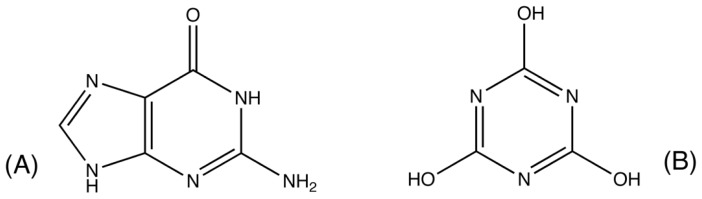
Structural comparison between (**A**) guanine (*i.e.*, the native substrate of human guanine deaminase), and (**B**) ammelide.

Having presented current computational enzyme redesign philosophies in great detail, we would like to only mention here some more noteworthy works to provide the interested reader with an overview of recent activity in the field. An area that has gained significant interest in recent years is enzyme specificity, as a result of increasing awareness [80–83] of the fact that, contrary to the classical image of enzyme catalysis, many (if not most) enzymes are catalytically promiscuous, catalyzing one or more chemically distinct reactions in addition to their native reaction. Significant effort has been invested into experimental redesign of enzyme specificity [84,85]. Along with this, inserting or modifying activity has also been the topic of recent computational studies. Earlier examples of this include Park *et al.*’s work [86], which introduced β-lactamase activity into a glyoxylase scaffold, while completely destroying the original activity. More recently, Korendovych and coworkers were able to computationally redesign calmodulin, which is a regulatory calcium binding protein, into an allosterically controlled Kemp eliminase that is activated upon Ca^2+^ binding by means of a single mutation [87]. Another recent work [88] used computational loop remodeling to control interactions between active site residues and the bound substrate and was able to alter the specificity of a human guanine deaminase to make it 100-fold more active with a structurally similar but chemically distinct substrate ammelide (Figure 7), and 2 × 10^4^-fold less active for the native guanine substrate, compared to the wild-type enzyme (resulting in a net specificity change of 2.5 × 10^6^-fold). A third significant recent work [89] focused on computationally redesigning a zinc-containing mouse deaminase to catalyze organophosphate hydrolysis (specifically the R(P) isomer of a coumarinyl analogue of the nerve agent cyclosarin), which, while giving only a modest initial phosphatase activity (*k*_cat_/*K*_M_ of 4 M^−1^ s^−1^), compared to the wild-type, which showed no detectable deaminase activity. However, this could be improved to a *k*_cat_/*K*_M_ of ~10^4^ M^−1^ s^−1^ after directed evolution. Therefore, rational enzyme redesign was again successfully used to provide a starting point for subsequent evolution to generate an enzyme with an activity comparable to that of an moderately efficient naturally occurring enzyme [20].

Finally, to conclude this section, we would like to briefly mention the increased interest in the challenging problem of designing efficient catalysts for chiral chemistry. Being able to control enzyme enantio- or regioselectivity is a problem of significant interest to the pharmaceutical industry, due to the role of chirality in drug efficacy, as well as potentially playing a major role in the industrial production of fine chemicals. However, computational redesign of stereo- or regioselecitvity poses a significant computational problem, due to the very small differences that govern selectivity as well as the tight dance between steric and electronic effects. There have been several computational attempts to address the origin of enzyme selectivity, however, these have often focused either on just ground states [90] or limited small models of the active site where the results can be very dependent on the precise starting structure used [91]. A more promising recent work [75] addressed the selectivity of the soluble epoxide hydrolase (sEH), which catalyzes epoxide ring opening to yield the corresponding vicinal diols. Through a combination of low-level QM/MM umbrella sampling molecular dynamics (MD) simulations and high-level *ab initio* calculations, the authors were able to rationalize the experimentally observed selectivity of the enzyme for phenyl *vs*. benzylic attack, identifying factors responsible for governing this selectivity. This work highlights the importance of proper sampling and examining multiple conformations, however, the obtained results only provided reasonable agreement with experimental rate constants when using a very high level of theory. Here, it should be noted that Frushicheva and Warshel recently examined the selectivity of wild-type and mutant *Candida antarctica* lipase using the EVB approach [92], and, as with earlier work on the Kemp eliminases [36,37], were able to obtain very high quantitative agreement with both the wild-type enzyme, as well as correctly reproducing (and rationalizing) the observed changes in selectivity upon mutation. It is worth highlighting here that this is a particularly challenging system that faces problems such as different degrees of water penetration, and, therefore, this work demonstrates the crucial importance of extensive sampling in order to obtain convergent results and to be able to reliably rationalize enantioselectivity [92].

## 4. *In Silico* “Directed Evolution”: Approaches to Reduce the Sequence Space in Laboratory Evolution Studies

Despite extensive improvements to rational enzyme design using computational approaches, the arguably most powerful tool in enzyme (re)design for biocatalytic purposes remains simply random (or semi-random) laboratory evolution [25,93]. Despite its power, the limitations in searching sequence space as well as the complexity of the enzymes’ catalytic actions remain inherent bottlenecks in all directed evolution studies. As a result of this, several experimental strategies are being developed to handle these problems in order to allow for high accuracy “low-throughput” screening (an issue discussed in detail in e.g., [94]). The question then arises of the extent to which computational approaches can contribute to this approach, guiding and rationalizing the ongoing experimental work. In light of the importance of this problem, it should not be surprising that there are also several computational strategies being developed in order to limit the search space in directed evolution studies as well as to perform screening of mutations *in silico*, allowing for a form of computational directed evolution. Here, we will address a number of these that can be used for improving enzymatic activity (many more of which have been reviewed elsewhere, see e.g., [95–98]). We aim to demonstrate that we are reaching a stage in the field where, rather than being limited to the early stages of artificial enzyme design or small-scale qualitative modifications, computational approaches can start to be used as a bridge across the gap that currently exists between rational design and laboratory evolution.

Early computational approaches towards reducing sequence space in DE studies included applying mean-field theory to explore protein fitness landscapes as well as the structural tolerance of individual residues [99], suggesting that mutations that are beneficial to activity and stability tend to occur at amino acid positions that are tolerant to substitutions. This was then matched by comparison to experimental directed evolution on subtilisin E and the T4 lysozyme, demonstrating that during experimental DE, favorable mutations were accumulated at the position predicted by this approach. A related approach is the SIRCH algorithm [100], which uses a second-order mean-field approach to identify residue-residue clashes from rotamer libraries, using atomistic representations to calculate rotamer-backbone, romater-intrinsic and rotamer-rotamer conformational energies. This can be used for truncating libraries in experimental directed evolution. More recent approaches for targeting directed evolution studies by exploring sequence function space include the protein sequence activity relationship algorithm (ProSAR) [101], currently owned by CODEXIS Inc™. This approach uses Kaufmann’s NK model [102] to describe the fitness landscape. Here, in this case N is the number of variable positions in the protein, and K is the degree of coupling between the variable positions. ProSAR then exploits a genetic algorithm to search the NK landscape, based on an iterative procedure in which a subset of protein sequences are generated from the full library of possible sequences. These are then ordered in terms of decreasing fitness, the top protein is retained as the backbone for the design work, and the list of residues at each position in the backbone is expanded by addition of subsequent protein sequences in decreasing order of fitness. This allows for the identification of residues potentially contributing most to fitness, and then parents are “mated” and the procedure is repeated iteratively until the desired property is observed (for more details, see [101]). A clear advantage of this approach in an industrial context is the limited knowledge of detailed chemistry that is required to achieve noticeable improvements in efficiency, and an example of a practical application of this approach was illustrated for instance in the 4000-fold improvement in the volumetric productivity of a cyanation process necessary for the synthesis of a cholesterol-lowering drug, atorvastatin (Lipitor), by a bacterial dehalogenase [103]. A few last examples include statistical coupling analysis (SCA), which is a bioinformatics procedure that uses sequence information to determine networks of energetically coupled co-evolving residues in proteins [104] and has already been applied to the design of allosteric communication in proteins [105] as well as the design of artificial sequences capable of folding to target structures [106]. Finally, while the SCA approach is powerful, an alternative approach based on evaluating energy-based allosteric matrices, which has already been successfully applied to explore the effect of mutations on transition state energetics and fidelity in DNA polymerases [107], is likely to be a powerful tool for not only the rational design of TSAs and drug design, but also for computational enzyme design.

Another issue that has gathered increasing attention recently is that of protein promiscuity, which has been hypothesized to play an important role in guiding enzyme evolution [80,81,83]. This is of potential importance to enzyme design [83], as reverse evolution to a promiscuous “progenitor” enzyme capable of catalyzing multiple reactions provides an attractive starting point for the insertion of novel functionality. Therefore, in addition to general interest in resurrecting ancestral proteins [108,109], there is also interest in mapping and redirecting enzyme evolutionary trajectories to modify enzyme functionality [110]. Finally, apart from standard bioinformatics-based approaches that can be used to do this, such as those harnessed in the aforementioned works, recent years have also seen a tendency to borrow from the social or economics sciences, using approaches such as Pareto efficiency or Pareto optimality [111] as an optimization procedure in protein design [112,113] for cases where there are multiple objects present, and an efficient and fast optimization procedure is needed.

**Figure 8:**
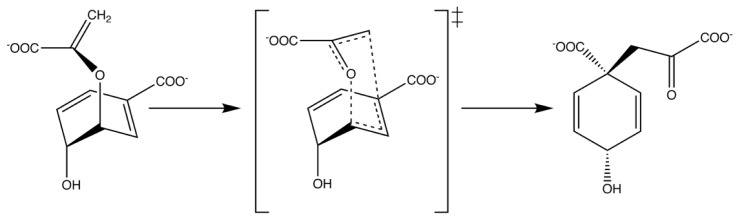
Overview of the mechanism for the rearrangement of chorismate to prephenate.

**Figure 9:**
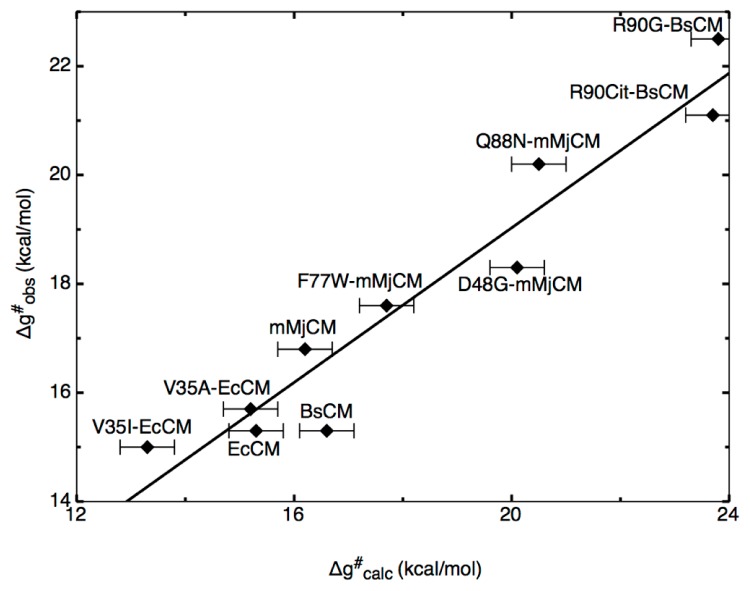
Correlation between calculated (Δg^≠^_calc_) and observed (Δg^≠^_exp_) activation barriers for the rearrangement of chorismate to prephanate catalyzed by the wild-type mono- (MjCM), di- (EcCM) and trimeric (BsCM) forms of chorismate mutase. The corresponding data for a range of mutants are also shown here. Note that R90Cit denotes a mutation to a non-standard amino acid (citrulline). Based on data presented in Table 2 of [114]. Calculations were performed using the empirical valence bond approach. In all cases, the calculated value is within ~2 kcal/mol of the experimental value.

A key common feature of the approaches described above is the fact that they rely on limited to no knowledge of the molecular details for the actual chemical step being catalyzed by the systems of interest. As the examples brought here highlight, there are cases where such approaches can rapidly provide improvements in enzyme activity, and are particularly useful in, for instance, industrial settings where a modest improvement at minimal cost is often more desirable than approaches that can provide better improvements but require far more input in terms of both manpower hours and chemical or computational expense. To conclude this section, we would like to highlight a number of recently developed screening approaches that directly target the chemical step, as well as the effect of mutations on subsequent folding stability, and highlight the fact that the gap between “black-box” approaches and those that require more insight into the actual system are rapidly decreasing allowing for effective targeting of actual chemistry and, therefore, potentially much higher improvements in catalytic efficiency than current approaches allow. As a starting point, considering the multitude of computational approaches that are currently available, it is important to focus on what is exactly required of a computational approach in order to be able to do effective computational enzyme design. As one of the main challenges with directed evolution studies is focusing the search space in an effective way, and the fact that it is hard to deduce the molecular basis for the beneficial or detrimental effects observed with different mutations, one would require from a computational approach that it can reliably reproduce catalytic effects in both wild-type and mutant enzymes (see also discussion in e.g., [114]). In such a way, it is possible to then use as a scoring function the expected effect of mutations on the catalytic effect in order to directly rank different constructs. A second issue, however, is the cost-to-benefit ratio of such an approach. Recent years have seen tremendous improvements in approaches for experimental directed evolution (see e.g., [94]), both increasing the library sizes that can be handled and also finding ways to focus the search space. Therefore, it is already possible to obtain quite impressive improvements on enzymatic activity using experimental approaches alone, and for a computational approach to be advantageous, it needs to provide an insight that the experiment cannot provide by itself, and to do so in a way that is faster than one can currently perform with experimental evolution. As discussed in Section 3, and illustrated by the data shown in Figure 6, we believe that one of the most effective ways of currently doing this is the EVB approach, as it not only allows you to rapidly explore mutational effects without the need for any further parameters adjustment once the reference state has been correctly calibrated, but also, it provides detailed breakdowns of the different contributors to this effect, allowing one to dissect the molecular basis for catalysis. In principle, one could do brute-force EVB to test different mutations (driven by chemical insight into the native reaction). However, considering the fact that it is necessary to do extensive sampling over multiple trajectories in order to get physically meaningful convergent results, computational cost will still become a limiting factor. Therefore, it can be useful to have a toolkit with which to do rapid initial screening of mutation hotspots prior to subsequent more detailed testing of the most promising constructs using EVB (and ultimately experiment). A number of these have been discussed and compared in detail in [114,115]. Specifically, these works compared the efficacy of different computational approaches for modeling the enzyme-catalyzed rearrangement of chorismate to prephenate (Figure 8), as well as for probing the effect of mutations on the observed catalytic activity of the different mono-, di- and trimeric forms of chorismate mutase (CM). This particular system was chosen as it has been important both for design studies [116], as well as for probing the validity of transition state analogues [64,116]. Specifically, the authors compared using the EVB directly to screen for mutations with focusing only on the TS electrostatic energy, using in this case the semimacroscopic version of the protein dipole Langevin dipole method, in its linear response approximation version (PDLD/S-LRA, for methodological details see [117]). The authors also explored the effectiveness of using the changes in EVB reorganization energies upon mutation as a screening tool. Here, it was found that, clearly, the EVB was the most effective tool for quantitative screening, due to its ability to reliably reproduce mutational effects in a quantitative manner (see e.g., Figures 6 and 9). In comparison, examining reorganization energies was found to be useful but suffered from convergence problems, and also, the PDLD/S-LRA (or the group contribution approach which dissects the contribution of different residues to the activation barrier or to TS binding energies) could be useful for identifying residues that can contribute to electrostatic stabilization of the TS, however, as it does not include the important effect of protein reorganization, it is mainly helpful as an initial screening approach to identify residues that could then be examined further by EVB.

The authors have since evolved and expanded on their screening approaches, highlighting their power as applied to the cases of the aforementioned Kemp elimination reaction [36,37] and also to an enantioselective lipase from *Candida Antarctica* [92] (CAL A). Here, the authors’ starting point is a simple and effective rapid screening approach, which bases itself on using the PDLD/S-LRA approach and estimating the electrostatic contribution of different residues (“groups”) to the activation barrier using the formulation:

(1) $$\Delta \Delta g_{elec}^{‡}≅332\sum_{ij}{\bar{q}}_{j}\Delta Q_{i}/r_{ij}{\bar{ɛ}}_{ij}$$

**Figure 10:**
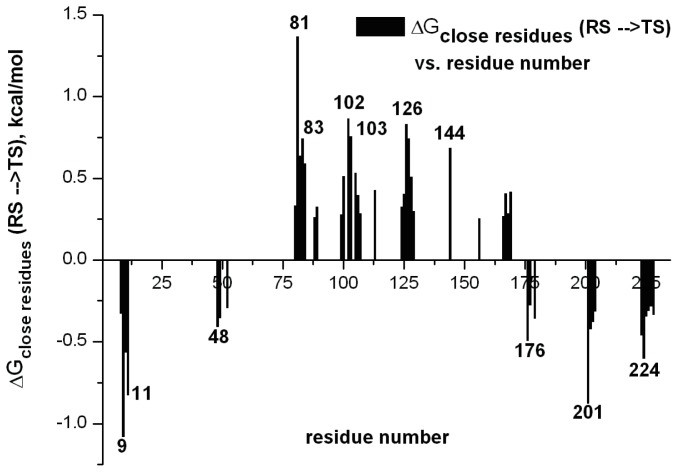
Predicting mutation hotpots for a designed Kemp eliminase. The y-axis denotes the interactions (in kcal/mol) between the protein residues with the substrate as its charge changes upon moving from the reactant state (RS) to the transition state (TS). Large negative contributions suggest optimal sites for mutations likely to enhance the catalytic effect. This figure was originally presented in [36].

Here, *q̄_j_* refers to the effective charge (or dipole in the case of polar residues) of the *q*^th^ residue, Δ*Q*_i_ refers to the change in substrate residual charges upon moving from the ground to the transition state, and ɛ*_ij_* represents the dielectric constant for a given interaction. The effect of mutating each residue can then be explored by artificially assigning a charge of 1.0 to all residues in the protein, and then identifying the charge change that will lead to the most negative ΔΔ*g*^‡^_elec_ (*i.e.*, the greatest stabilization upon mutation). An example of this in the case of a designed Kemp eliminase [36] is shown in Figure 10.

This then leads to the formulation:

(2) $${\left(\Delta {\bar{q}}_{j}\right)}_{opt}≅-\alpha \partial \Delta \Delta g_{elec}^{‡}/\partial q_{j}=-\alpha \sum_{i}\Delta Q_{i}/r_{ij}{ɛ}_{ij}$$

where the optimal values of Δ*q̄_j_* are proportional to the electrostatic group contributions in the case when all the protein groups are positive charges, and α is a proportionality constant. Such an approach is particularly useful in cases where the effect is predominantly electrostatic, and also for challenging cases where the charge change upon moving from ground to product state is sufficiently small to makes it hard to directly exploit the polarity of the active site residues (see [36]). However, it can be insufficient in cases such as enzyme enantioselectivity, where the observed effect is due to not only electrostatic but also steric effects, by combining the linear response approximation (LRA) approach [118], which can provide a reliable estimate of the free energy associated with changing the electrostatic potential of the system from one potential to another, with the steric (nonelectrostatic) component of Åqvist’s linear interaction energy (LIE) approach [119,120], which introduces an empirical parameter (β) that scales the van der Waals component of the protein-ligand interaction. Such a combined LRA/β approach has shown significant promise in the test case of CAL A [118]. A final critical issue is, of course, the effect of such mutations on protein stability. That is, stability should minimally not be significantly impaired, and ideally retained or even optimized. This can be done in an effective way using a focused dielectric constant [121], which approximates the folding energy using the expression:

(3) $$\Delta G_{fold}≅332\sum_{ij}q_{i}q_{j}/r_{ij}{ɛ}_{focus}$$

Here, ɛ_focus_ denotes the optimal dielectric constant for a given interaction, and *q*_i_ and *q*_j_ are the charges of the relevant ionized residues at a given pH [121]. For other recent examples of predicting protein stability upon mutation using computational approaches we refer the reader to [122–126], amongst others. As discussed in e.g., [37], combining approaches to optimize TS stabilization with protein stability constraints can provide a highly efficient approach to predict optimal mutations of particularly distant (from the active site) ionized residues, and, as can be seen, while this is an area still in its infancy, significant progress is being made at a rapid pace, and we believe that approaches such as these will be the most effective currently available in the move towards *in silico* directed evolution.

## 5. How do Enzymes Actually Work, or Why is Computational Enzyme Design so Difficult?

Computers are constantly increasing in power, and, in theory, computational approaches should play a major role in the design of novel catalysts. However, as illustrated in Sections 2 and 3, despite impressive advances in computational enzyme design (both *de novo* and by redesigning existing systems), the actual rate enhancements obtained by the designed systems are quite poor, and clearly far from that of naturally occurring systems. The question then becomes why this should be the case? This has been a topic of significant discussion [36,37,66,116], and suggestions as to the source of this problem have included, at the most qualitative level, simply that either the active site construct is incorrect (based on the argument that enzymes require idealized active sites), that the idealized active site is not realized in practice, and that the designed active site is not supported by the scaffold it is placed into [66].

While valid points, such arguments reflect the focus that has been placed on achieving shape complementarity in many of the enzyme design studies discussed in this work. Here, we would like to argue that the actual situation is more complex than this. That is, enzymes are chemical catalysts, and therefore, in order to be able to engage in effective enzyme (re)design, it is important to have detailed insight into how enzymes actually work at the molecular level. Indeed, being able to design effective artificial enzymes is perhaps one of the best proofs that we have finally solved this problem. However, the question of how enzymes actually work is one that has eluded enzymologists for over a century, and multiple hypothesis have been put forward to try to rationalize the tremendous catalytic proficiencies of enzymes, including desolvation effects, strain, and entropy loss during the reaction coordinate (see discussion in e.g., [13,127,128]). The problems with these (and related) hypotheses have been discussed in detail elsewhere [13,70,129,130]. However, we would like to focus on a hypothesis that has gained a lot of popularity in recent years, namely the potential importance of dynamical effects to enzyme catalysis [131–135]. One of the problems with this hypothesis is that it has not been clearly defined: That is, clearly, once atoms are above a few degrees K they move, and enzymes are dynamic entities. Additionally, many enzymes utilize conformational modification as an important part of their catalytic cycles. However, whether such dynamics can actually contribute to the *chemical* step of enzyme catalysis is a different issue, and one that is not possible to test experimentally, as there is no experiment that can actually check this. Therefore, arguments in favor of the dynamical hypothesis have been mainly based on *indirect* observations such as similar timescales for the conformational and chemical steps [132,133]. We explored this issue in great detail [128] and demonstrated that, based on our simulations, this would strongly suggest that the chemical step has no memory of any preceding conformational transition [136]. Additionally, even anticatalytic mutations that apparently provide direct evidence for the importance of dynamics in enzyme catalysis [134] were demonstrated to be electrostatic in origin [77]. Other workers than us have also expressed concern over this issue [137–139]. We bring this up here due to the increasing popularity of invoking dynamical effects as being important for artificial enzyme design (see e.g., [140]). Clearly, conformational changes can be very important for enzyme function, and by modulating such changes one also modulates the activity of the enzyme. However, as one is only likely to impair the catalytic activity of the enzyme through this, and as the enzyme appears to have no memory of the conformational change once the chemical step has started, it is unclear how such effects are likely to actually aid enzyme design.

**Figure 11:**
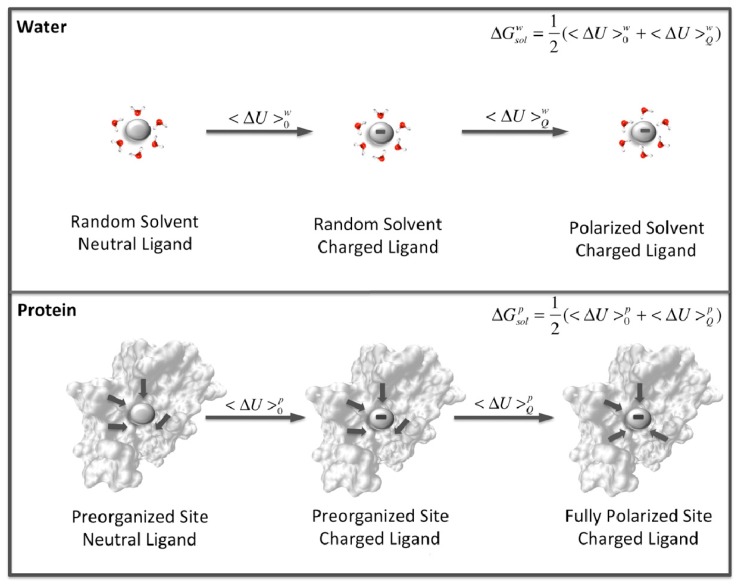
Schematic overview of the preorganization effect. This figure first considers the charging of a substrate in an environment that has not been polarized by the substrate and then illustrates the effect of the polarization of the solvent by the field of the substrate, which is substantially larger in (**A**) water than in (**B**) protein. This figure was originally presented in [65].

While it is unlikely that dynamical effects play an important role in the actual rate acceleration that enzymes have evolved to optimize, a number of other factors clearly are to different degrees. These include well-characterized chemical effects such as acid-base catalysis and covalent catalysis, both of which can account in relevant cases for some of the observed catalytic effect. The major contributor, however, as has been observed by countless simulation studies [13], is the electrostatic preorganization of the active site [141] (Figure 11). Specifically, the reorganization penalty associated with reorienting randomly oriented water dipoles as the environment becomes polarized by changing charge on the substrate is much higher than the corresponding effect in an enzyme active site, where the relevant dipoles and charges are already optimally organized. The importance of electrostatic complementarity has been particularly highlighted in the case of ketosteroid isomerase [65,142,143], which has been one of the “classical” systems to challenge the electrostatic preorganization idea [144–146], and where it was demonstrated that almost the entire origin of the catalytic effect is electrostatic [65]. Now the fact that an increasing number of enzymes are being demonstrated to be “catalytically promiscuous” [83], catalyzing multiple, chemically distinct transition states in addition to their native transition state, with proficiencies that can at times almost compete with that for their native reaction [147–150], would superficially appear to be at odds with the idea of electrostatic preorganization (and the argument above that enzymes have “idealized” active sites). However, a recent computational work (to the best of our knowledge) that has comparatively explored both structural and electrostatic features driving catalysis of multiple substrates in a quantitative fashion in an extremely promiscuous arylsulfatase [151,152] has demonstrated that the enzyme is able to identify and distinguish between different, chemically distinct transition states, but that the promiscuity is simply driven by the ability of the promiscuous substrates to exploit the pre-existing electrostatic preorganization of the active site for the native substrate. Similar qualitative observations were made for a related system based on experimental work [153]. Therefore, the promiscuity appears to even in such a case be electrostatically-based [152], suggesting a role for chemistry-driven protein evolution. It is also worth noting that in this study, the two best substrates examined (out of four) proceeded through the most compact and expansive transition states, respectively. Also, out of a number of promiscuous substrates, the one that the enzyme preferentially catalyzed was the largest and bulkiest of these (in agreement with experimental data [147]), illustrating that the enzyme can exploit its active site plasticity to adapt to the substrate if the electrostatics match. Therefore, it could be argued that, rather than focusing on shape complementarity, it would be much more effective to focus on electrostatic complementarity, and use rational chemistry-driven protein redesign in order to obtain effective catalysts (see also discussion in [36,37]).

## 6. Conclusions and Future Perspectives

Artificial enzymes have the potential to play a major role in sustainable development, providing green reusable catalysts for processes encompassing all aspects of life, from generating new therapeutics to the food industry to their use as detergents. Therefore, interest in using enzymes as artificial catalysts has exploded, and biocatalysis is one of the most rapidly growing current fields (see e.g., [15,34,154,155]). However, design of artificial enzymes requires an intimate understanding of how enzymes work, and to date, the precise molecular details of enzyme catalysis still remain controversial and to some extent elusive, although electrostatics clearly plays a central and dominant role [13]. Therefore, any progress in artificial enzyme design will be aided by parallel progress in our understanding of enzymology, and the ability to effectively design artificial enzymes will in turn be the best proof that we have finally understood how enzymes work.

Despite these challenges, recent years have seen significant progress in both *de novo* computational enzyme design, from minimal active site models, as well as computational protein redesign, based on existing templates, and constant increases in computational power are expected to continue to accelerate such advances. Nevertheless, impressive as such studies have been, as has been illustrated by the examples brought up in this review that there is still a very large gap between the proficiencies of natural and designed enzymes. Many arguments have been put forward to rationalize this [66,116], some of which have focused on the importance of having an idealized active site and the problems with realizing such a situation. However, as was outlined in Section 5, many promiscuous enzymes do not have idealized active sites, recognizing multiple chemically distinct transition states (e.g., [147–150,153]), with often quite high efficiency. Here, in the only example known to us where such promiscuity has been *quantitatively* dissected using computational approaches to compare the catalysis of multiple substrates, it appeared that the main driving force for the promiscuity was simply the electrostatic preorganization of the active site, which, while it is optimized for the native substrate, can also be flexible enough to accommodate multiple, chemically distinct substrates [152]. If it can be extended to other systems, such electrostatic flexibility, whether naturally occurring or engineered, can clearly be exploited for enzyme design. This then raises a number of questions. The first is that of what the best starting point actually is for enzyme design. That is, while clearly the ability to engineer novel catalysts completely *de novo* is impressive in and of itself, Nature already provides a vast range of templates for many different types of chemistry, and naturally occurring enzymes are evolving all the time. Therefore, we believe that there is a strong argument in favor of starting from a naturally occurring system that demonstrates evolvability and manipulating its activity, rather than performing completely *de novo* enzyme design. However, regardless of the starting point, the next issue becomes that of how to effectively improve such systems. As was illustrated in Section 2, even in cases where the initial catalytic activities of *de novo* designed systems were relatively poor, subsequent rounds of experimental evolution can improve on such poor activities. Despite its power, however, directed evolution studies will inherently be limited by the vastness of the sequence space combined with low frequencies of desirable mutations even in targeted libraries [26]. In light of this, we are therefore at a very exciting time in the field. As illustrated in Section 4, recent years have seen substantial advances in approaches to reduce the search space needed in directed evolution studies, but also, even more importantly (in light of the fact that enzymes are chemical catalysts), in approaches that can effectively and reliably screen for mutation hotspots and predict the effect of mutations *in silico*, even prior to any experimental testing. Such approaches can directly target the chemical step, dissecting the contribution of different residues to catalysis, and quantifying the effect of modification of different residues both directly in the active site and even quite far from it. Critically, such approaches (such as, e.g., those discussed in [36,37,114,115,118]) allow for extensive sampling and take into account the reorganization of the protein environment, which is often missing in many design studies. This means that they can be used to study complex problems such as that of enzyme selectivity [118], where a very subtle balance between steric and electrostatic effects can completely determine the preference of an enzyme for one form of the substrate, or position of attack, over another. Clearly there are still challenges involved in such chemistry-driven approaches, which include the problems of dealing with systematic searches of the effect of multiple mutations simultaneously. Now this problem can, for example, be alleviated by use of a coarse-grained model that can be extended to examine multiple mutants at once, while then also extrapolating back to an all-atom model, as discussed in [118]). More critical, however, is the risk that incorporated mutations could cause large-scale conformational rearrangements, or even collapse, that are not captured by the computational approach (although many approaches for predicting protein stability upon mutation are currently being developed, with varying degrees of success [121–126]). In the long run, once such approaches can be coupled with approaches that can reliably predict the effect of mutations on enzyme physico-chemical properties such as thermal stability and solubility, this would then provide an ideal starting point for experimental testing. Such an iterative approach, in which the experimental evolution is guided by rational *in silico* evolution (which is in turn guided by detailed knowledge of the molecular machinery for catalysis), with theory being used to guide and rationalize experiment and experiment being used to test, validate and refine theory, will ultimately allow for a far more efficient design strategy. This, in addition to accelerating the design process overall by allowing a significant part of it to be performed computationally, also accelerates the design process by allowing for the construction of focused “smart” libraries for experimental evolution, significantly reducing the size of the sequence space that needs sampling. When this is combined with parallel advances in *de novo* enzyme design and structure-based protein redesign, this will provide a much-needed bridge, which closes the gap that currently exists between computational enzyme design and laboratory evolution.
